# Impact of anatomical changes on simulated proton arc therapy and intensity‐modulated proton therapy for lung cancer

**DOI:** 10.1002/acm2.70881

**Published:** 2026-10-26

**Authors:** Wenguang Chen, Feng Yang, Fanrui Zeng, Hao Guo, Yuankang Xu, Tianyu Zhang, Junxiang Wu, Da Zhang, Gang Liu, Xuanfeng Ding, Xianliang Wang

**Affiliations:** ^1^ Applied Nuclear Technology in Geosciences Key Laboratory of Sichuan Province Chengdu University of Technology Chengdu China; ^2^ Department of Radiation Oncology Sichuan Clinical Research Center for Cancer Sichuan Cancer Hospital & Institute, Sichuan Cancer Center, School of Medicine, University of Electronic Science and Technology of China Chengdu China; ^3^ School of Physics University of Electronic Science and Technology Chengdu China; ^4^ Institute of Nuclear Science and Technology, Sichuan University Chengdu China; ^5^ Cancer Center, Union Hospital, Tongji Medical College, Huazhong University of Science and Technology Wuhan China; ^6^ Department of Radiation Oncology, Corewell Health William Beaumont University Hospital Royal Oak Michigan USA

**Keywords:** anatomical changes, intensity‐modulated proton therapy, lung cancer, proton arc therapy

## Abstract

**Purpose:**

: During lung cancer radiotherapy, inter‐fractional anatomical changes may cause the delivered dose to deviate from the primary plan, affecting tumor control and increasing the risk of normal tissue damage. This study aims to evaluate the impact of anatomical changes on the dosimetric and radiobiological performance of simulated proton arc therapy (sPAT) versus intensity‐modulated proton therapy (IMPT) for lung cancer patients under patient‐specific anatomical changes.

**Methods:**

: Data from 21 lung cancer patients were retrospectively analyzed. The prescribed dose for all patients was 6000 cGy (relative biological effectiveness, RBE). Each patient with a primary planning computed tomography (pCT) and a mid‐treatment adaptive‐planning CT (aCT) acquired at a median of 29 days into treatment due to notable tumor volume changes. Primary plans for sPAT and IMPT were designed on the pCT. After rigid registration of the pCT and aCT, non‐adaptive plans (sPAT_p‐a_, IMPT_p‐a_) were generated by directly recalculating the doses of the primary plans on the aCT. The comparison evaluated the dosimetric impact of anatomical changes by comparing non‐adaptive plans (sPAT_p‐a_ vs. IMPT_p‐a_). Dose‐volume histogram (DVH) parameters for targets and organs at risk (OARs) and tumor control probability (TCP) and normal tissue complication probability (NTCP) were evaluated.

**Results:**

: For the primary plans, sPAT and IMPT achieved similar target coverage, while the doses to OARs were generally lower in sPAT_p_. Under anatomical changes, both non‐adaptive plans exhibited a decrease in target coverage, sPAT_p‐a_ achieved a D_95%_ of 5567.57 cGy[RBE] compared with 5423.33 cGy[RBE] for IMPT_p‐a_ (*p* < 0.001), indicating that sPAT better preserved target coverage. Compared with the primary plans, sPAT_p‐a_ and IMPT_p‐a_ exhibited TCP reductions of 5.83% and 8.07%, respectively.

**Conclusions:**

: Compared with IMPT, sPAT exhibits enhanced robustness against inter‐fractional anatomical changes occurring in lung‐cancer radiotherapy.

## 1 INTRODUCTION

Proton therapy exploits the characteristic Bragg‐peak dose‐deposition property of proton beams to achieve effective tumor ablation while limiting dose deposition in adjacent normal tissues, conferring theoretical dosimetric advantages relative to conventional photon‐based radiotherapy.^1^ Nevertheless, intensity‐modulated proton therapy (IMPT) remains susceptible to density variations along beam paths induced by setup uncertainties, intra‐ and inter‐fractional anatomical changes. Such perturbations may degrade delivered dose distributions, leading to insufficient target coverage or excessive irradiation of organs‐at‐risk (OARs).^2^, ^3^

Proton arc therapy (PAT) leverages gantry rotation to deliver irradiation across multiple beam angles. By spatially dispersing uncertainties associated with individual beam directions, PAT can theoretically yield more homogeneous target dose distributions and mitigate localized high‐dose burdens to OARs.^4^ While prior investigations have contrasted baseline dosimetric performance between PAT and IMPT under robust‐optimization conditions,^5^, ^6^ few studies have systematically evaluated their robustness in the setting of patient‐derived inter‐fractional anatomical modifications.

Compared to conventional fixed‐field proton therapy, PAT is expected to demonstrate greater robustness against anatomical changes. Lung cancer patients were selected as the focus of this study for the following reasons: (1) substantial tumor shrinkage is frequently observed during conventionally‐fractionated radiotherapy, with volume reductions often exceeding 20%–40%; (2) the large density difference between lung parenchyma and soft tissue/tumor creates dramatic interfaces that amplify proton range perturbations when anatomy changes; and (3) anatomical changes including tumor regression, changes in atelectasis, and the appearance or resolution of pleural effusion are frequently observed and well‐documented.^7^, ^8^

In this study, we implemented simulated PAT (sPAT) using a discrete‐beam, static pseudo‐arc approach and systematically compared its dosimetric and robustness differences with IMPT under patient‐specific anatomical changes. By generating non‐adaptive, we assessed dose degradation under conditions of observed anatomical change captured on mid‐treatment repeat CT. Dose changes caused by anatomical changes may further affect clinical endpoints such as tumor control and normal tissue toxicity. To reflect these potential effects, we evaluated the clinical impact through tumor control probability (TCP) and normal tissue complication probability (NTCP).

## 2 MATERIALS AND METHODS

### 2.1 Patient selection

This study was a retrospective dosimetric comparative analysis. Twenty‐one pathologically‐confirmed lung‐cancer patients who received conventional fractionated photon radiotherapy (60 Gy in 30 fractions) between January 2024 and July 2025 were included. Enrolment was restricted to patients exhibiting notable reduction in tumor volume who underwent clinically‐indicated mid‐treatment re‐simulation adaptive CT (aCT). The aCT scan was acquired at a median interval of 29 days following the primary‐planning CT (pCT). Patient demographic and tumor‐volume data are summarized in Table 1. Declines in clinical‐target‐volume (CTV) from median 31.99 cm^3^ (pCT) to 18.01 cm^3^ (aCT) reflect anatomical and density modifications that can perturb proton range and resultant dose distributions.

**TABLE 1:** Characteristics of the patients.

Characteristics		No.
Sex	Male	18
	Female	3
T stage	T1	2
	T2	4
	T3	6
	T4	9
	Median	Range
Age (years)	65	59–70
CT interval time (days)	29	20–34
CTV (pCT, cm^3^)	31.99	17.94–56.56
CTV (aCT, cm^3^)	18.01	11.55–43.28

Abbreviations: CTV, clinical target volume; pCT, primary planning CT; aCT, adaptive planning CT.

### 2.2 Treatment planning

Both the pCT and aCT were average‐intensity‐projection CT images reconstructed from 4DCT datasets acquired during free breathing, representing the time‐averaged anatomy and mitigating respiratory motion artifacts. For each patient, primary plans (sPAT_p_ and IMPT_p_) were optimized on the pCT first. For the non‐adaptive plans, the CTV and OARs on the aCT were manually re‐delineated by a radiation oncologist to reflect the updated anatomy at the time of the repeat scanning. After rigid image registration of pCT onto aCT, primary‐plan beam configurations and spot weights were transferred and dose was recalculated on aCT without further plan optimization to produce non‐adaptive plans.

All proton therapy plans were developed using RayStation (version 7.0, RaySearch Laboratories, Stockholm, Sweden). To eliminate inter‐planner variability, all treatment plans in this cohort were generated by a single medical dosimetrist. For IMPT plans, 3–4 beams were individually selected for each patient based on the specific tumor location and surrounding OARs, employing multi‐field optimization (MFO).

sPAT was implemented using a discrete‐beam, static pseudo‐arc approach, consistent with previously reported static arc‐like proton planning.^9^ This implementation was an idealized, delivery‐unconstrained, discrete‐beam pseudo‐arc approximation. For each patient, beams were placed at 10° intervals, and the angular range was individually adapted to the patient's tumor geometry, spanning 120°–160° of an ipsilateral arc and yielding 13–17 beams per plan. Full 360° rotation was deliberately avoided so that no beam traversed the contralateral healthy lung. All beams and all their energy layers and spots were optimized simultaneously within a single MFO; no upper limit was imposed on the number of energy layers per beam, and the optimizer autonomously selected energy layers to satisfy target and OARs objectives. The essential difference between sPAT and multi‐field IMPT does not lie in the delivery technique but in the angular sampling: both are static fixed‐field plans, and neither simulates dynamic arc delivery. IMPT used a few clustered fixed beam directions, whereas sPAT used more numerous, denser beam directions distributed along an ipsilateral arc. Therefore, sPAT is technically equivalent to a highly multi‐field IMPT plan with dense, arc‐like angular sampling; if IMPT adopted the same angular sampling as sPAT, the two would define the same optimization problem and differ only in name. Here, “arc” describes only the angular distribution of the sampled beam directions, not a continuous dynamic delivery trajectory.

Dose calculation was performed using the Monte Carlo algorithm with statistical uncertainty set to 0.5%. CTV‐based robust optimization incorporated 3 mm setup‐shift uncertainty and 3% proton‐range uncertainty, parameters consistent with widely‐accepted proton‐therapy planning guidance for lung‐cancer cases.^10^ A total of 21 robust‐evaluation scenarios were computed: 1 nominal scenario, 6 setup‐shift scenarios, 2 range‐error scenarios, and 12 combined setup‐ and range‐error scenarios.

The relative biological effectiveness (RBE) factor was fixed at 1.1. All patients were prescribed a dose of 6000 cGy[RBE]/30 fractions. The planning objective required 95% of the CTV volume to receive prescription dose. OARs dose constraints were established according to the treatment planning guidelines recommended by the Particle Therapy Co‐Operative Group (PTCOG) and the conventional fractionated proton therapy OARs limits for lung cancer from the MD Anderson Cancer Center.^11^, ^12^

### 2.3 Plan evaluation

Dosimetric evaluations were performed on dose‐volume histogram (DVH) generated from the nominal dose distributions of the sPAT and IMPT. All primary‐plan dose distributions were normalized such that CTV D_95%_ equaled 6000 cGy[RBE]. Target coverage parameters included the dose covering 98% of the target volume (D_98%_), the dose covering 95% of the target volume (D_95%_), and the dose covering 2% of the target volume (D_2%_). Evaluated OAR parameters encompassed lung V_5_, lung V_20_, lung mean dose, esophagus mean dose, heart mean dose, esophagus maximum dose, and spinal‐cord maximum dose. Conformity index (CI)^13^ and homogeneity index (HI)^14^ were computed using the following definitions:

$$\text{CI}=\left(V_{\mathrm{Tref}}/V_{T}\right)\times \left(V_{\mathrm{Tref}}/V_{\mathrm{ref}}\right)$$

where V_Tref_ is the target volume receiving at least the reference (prescription) dose, V_T_ is the total target volume, and V_ref_ is the total volume receiving at least the reference dose. A CI value closer to 1 indicates better dose conformity.

The HI was calculated as:

$$\text{HI}=\left(D_{2\%}-D_{98\%}\right)/D_{50\%}$$

where D_50%_ is the dose covering 50% of the target volume. A HI value closer to 0 indicates better dose homogeneity.

To quantify the dosimetric impact of anatomical changes, dose differences (ΔX = X_non‐adaptive_−X_primary_, where X denotes a DVH parameter) were calculated for corresponding non‐adaptive and primary plans.

### 2.4 TCP and NTCP calculation

Radiobiological outcomes were assessed with tumor‐control probability (TCP) and normal‐tissue‐complication probability (NTCP). TCP was derived by converting heterogeneous DVH data into equivalent‐uniform‐dose (EUD) values using the Niemierko model.^15^, ^16^ NTCP were computed using the Lyman–Kutcher–Burman (LKB) model.^17^ All model parameters were derived from previously published literature and kept consistent across all radiotherapy plans; the specific parameters are summarized in Table 2.

**TABLE 2:** Parameters for the TCP and NTCP calculation.

	Structure	TCD_50_/TD_50_ (Gy)	γ50/m	a/n	Endpoint
TCP	CTV	51.24	0.83	−10.00	Tumor control^17^
NTCP	Lung	24.50	0.18	0.87	Pneumonitis^18^
	Heart	48.00	0.10	0.35	Pericarditis^18^
	Esophagus	51.00	0.32	0.44	Esophagitis^19^
	Spinal Cord	66.50	0.18	0.05	Myelitis/Necrosis^20^

Abbreviations: NTCP, normal tissue complication probability; TCP, tumor control probability.

### 2.5 Statistical analysis

Statistical analyses were performed using SPSS software (v26.0, IBM Corp, Armonk, NY, USA). The Shapiro‐Wilk test was used to assess normality of pairwise‐difference distributions. When pairwise differences satisfied normality (Shapiro‐Wilk *p* > 0.05), paired‐sample *t*‐test was applied; otherwise, the two‐tailed Wilcoxon signed‐rank non‐parametric test was used. A threshold of *p* < 0.05 was considered statistically significant. All results are presented as mean ± standard deviation (*x̄* ± SD).

## 3 RESULTS

### 3.1 Representative dose distributions

Figure 1 displays dose distributions for one illustrative representative patient for visual demonstration. Quantitative conclusions are drawn from the cohort‐level analyses in subsequent sections. Both primary plans achieve comparable target coverage (see Figure 1a,c). Following anatomical changes simulated within non‐adaptive recalculations, the prescription‐dose region in IMPT_p‐a_ no longer adequately overlapped with target anatomy, producing evident target‐coverage loss. In contrast, sPAT_p‐a_ maintains comparatively preserved target coverage (Figure 1b,d). Corresponding DVH comparisons are presented in Figure 1e,f.

**FIGURE 1:**
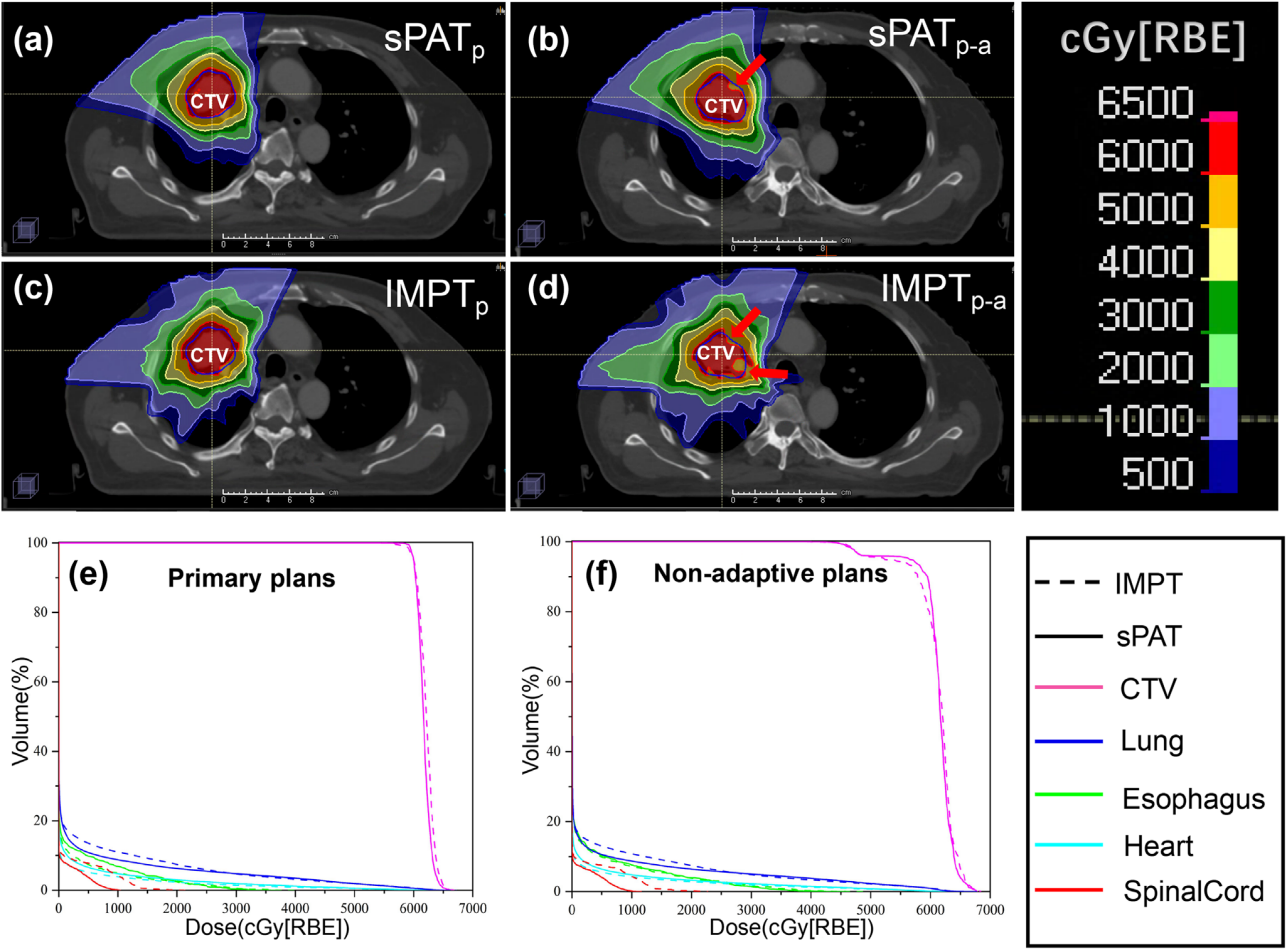
Dose distributions for one illustrative representative patient. (a–b) Dose distributions for sPAT in the primary and non‑adaptive plans. (c–d) Dose distributions for IMPT in the primary and non‑adaptive plans. (e–f) DVH comparisons between sPAT (continuous line) and IMPT (dashed line) for the primary and non‑adaptive plans.

### 3.2 Dosimetric evaluation of primary plans

The dosimetric parameters are summarized in Table 3. Both sPAT_p_ and IMPT_p_ achieve comparable target coverage (D_95%_ normalized to 6000 cGy[RBE]). The D_2%_ of IMPT_p_ is slightly higher than that of sPAT_p_ (*p* = 0.001), and CI and HI are both superior in sPAT_p_ compared to IMPT_p_ (*p* < 0.05). For OARs, sPAT_p_ demonstrates significantly lower lung V_20_ and V_5_ (*p* < 0.001), and notably, the spinal cord D_max_ in IMPT_p_ is on average 415.61 cGy[RBE] higher than in sPAT_p_ (*p* = 0.001). The esophageal D_mean_ in sPAT_p_ is slightly higher than that in IMPT_p_, whereas the heart D_mean_ is marginally lower; however, these differences are not statistically significant.

**TABLE 3:** Dose‐volume histogram parameters for primary plans.

		Primary plans		
Structure	DVH parameters	sPAT_p_ (*x̄* ± SD)	IMPT_p_ (*x̄* ± SD)	*p* value
CTV	D_98%_(cGy[RBE])	5945.67 ± 33.19	5930.33 ± 30.17	0.118
	D_95%_(cGy[RBE])	6000.00 ± 0.00	6000.00 ± 0.00	1.000
	D_2%_(cGy[RBE])	6367.00 ± 74.89	6450.29 ± 89.89	0.001^*^
	CI	0.56 ± 0.11	0.54 ± 0.11	0.002^*^
	HI	0.07 ± 0.01	0.08 ± 0.02	<0.001^*^
Lung	V_20_(%)	5.96 ± 1.98	6.55 ± 2.02	<0.001^*^
	V_5_(%)	10.77 ± 3.59	12.12 ± 3.44	<0.001^*^
	D_mean_(cGy[RBE])	309.05 ± 89.16	332.86 ± 87.19	0.010^*^
Esophagus	D_mean_(cGy[RBE])	148.90 ± 248.54	144.38 ± 250.24	0.372
	D_max_(cGy[RBE])	1886.57 ± 2188.90	1975.00 ± 2214.74	0.123
Heart	D_mean_(cGy[RBE])	70.90 ± 85.58	72.10 ± 99.49	0.214
Spinal Cord	D_max_(cGy[RBE])	570.86 ± 575.30	986.47 ± 951.52	0.001^*^

*Note*: All DVH parameters are presented as mean ± standard deviation (*x̄* ± SD).

* indicates a statistical significance at *p* < 0.05.

### 3.3 Impact of anatomical changes on dosimetry

The dosimetric parameters for the non‑adaptive plans are summarized in Table 4. Anatomical changes, primarily tumor regression, induced dose degradation for both non‐adaptive plan techniques. sPAT_p‐a_ preserved superior CTV D_98%_ and D_95%_ compared to IMPT_p‐a_. CI and HI parameters remained more favorable for sPAT_p‐a_. For OARs, sPAT_p‐a_ maintained lower lung V_20_, V_5_, esophageal D_max_ and spinal‐cord D_max_; spinal‐cord D_max_ for sPAT_p‐a_ was approximately half of IMPT_p‐a_. No statistically‐significant inter‐technique differences were observed for esophageal and heart D_mean_ among non‐adaptive plans.

**TABLE 4:** DVH parameters for non‐adaptive plans.

Structure	DVH parameters	sPAT_p‐a_ (*x̄* ± SD)	IMPT_p‐a_ (*x̄* ± SD)	*p* value
CTV	D_98%_(cGy[RBE])	5275.71 ± 767.77	5137.71 ± 777.98	0.005^*^
	D_95%_(cGy[RBE])	5567.57 ± 579.38	5423.33 ± 642.29	<0.001^*^
	D_2%_(cGy[RBE])	6390.33 ± 132.54	6502.05 ± 107.47	<0.001^*^
	CI	0.34 ± 0.16	0.32 ± 0.15	0.005^*^
	HI	0.18 ± 0.14	0.22 ± 0.13	<0.001^*^
Lung	V_20_(%)	6.02 ± 2.03	6.60 ± 2.01	<0.001^*^
	V_5_(%)	11.22 ± 3.69	12.58 ± 3.50	<0.001^*^
	D_mean_(cGy[RBE])	317.10 ± 92.98	340.19 ± 89.85	<0.001^*^
Esophagus	D_mean_(cGy[RBE])	164.33 ± 290.82	152.71 ± 246.27	0.687
	D_max_(cGy[RBE])	1920.48 ± 2095.64	2129.24 ± 2112.37	0.017^*^
Heart	D_mean_(cGy[RBE])	91.86 ± 127.39	92.90 ± 139.19	0.406
Spinal Cord	D_max_(cGy[RBE])	665.95 ± 701.31	1224.52 ± 1137.44	<0.001^*^

*Note*: All DVH parameters are presented as mean ± standard deviation (*x̄* ± SD).

* indicates a statistical significance at *p* < 0.05.

Dose differences relative to the primary plans are illustrated in Figure 2. Magnitudes of target‐coverage loss are substantially larger for IMPT (*p* < 0.05). The most pronounced OARs dose difference is observed in spinal cord D_max_, where the Δ_Dmax_ for IMPT is 2.5 times that of sPAT. For other OARs (lung, esophagus, and heart), the dose differences between the two techniques are comparable (*p* > 0.05).

**FIGURE 2:**
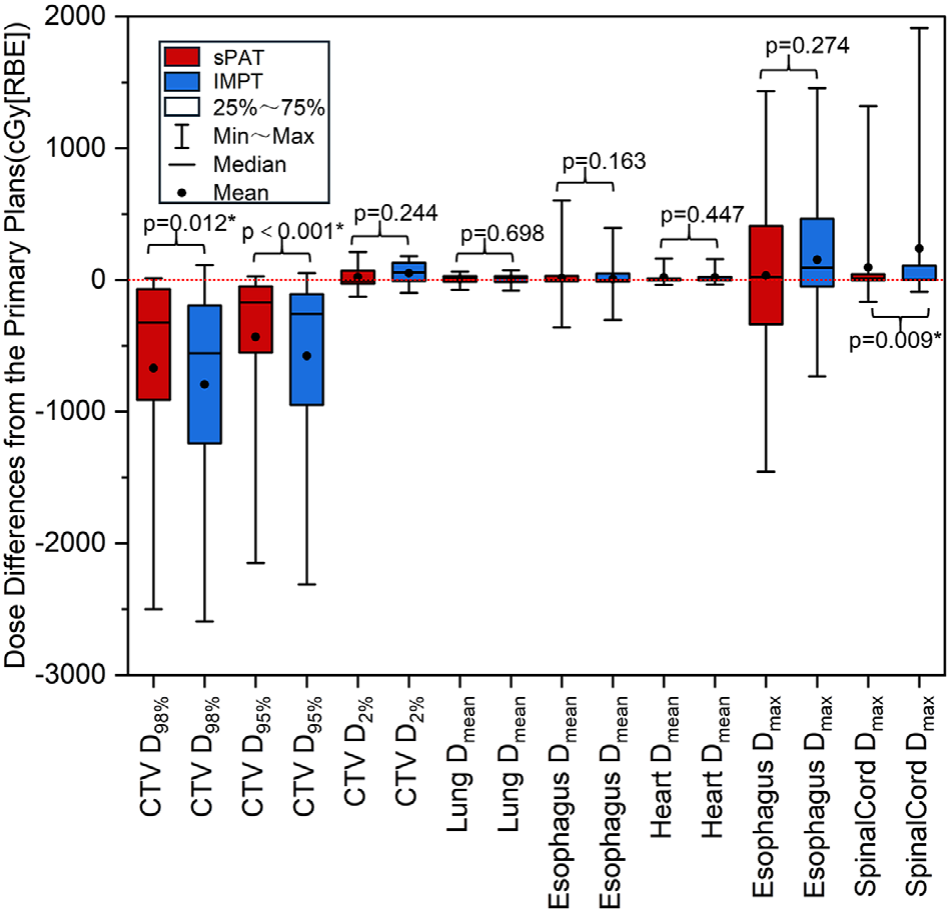
Dosimetric differences between non‑adaptive and primary plans for sPAT (red) and IMPT (blue). * indicates a statistical significance at *p* < 0.05.

### 3.4 TCP and NTCP evaluation

TCP and NTCP results are summarized in Table 5. In the primary plans, the mean TCP values are 64.99% for sPAT_p_ and 65.58% for IMPT_p_. Following anatomical change modelled within non‐adaptive recalculations, TCP substantially decrease for both techniques, with average reductions of approximately 5.83% for sPAT and 8.07% for IMPT; for most OARs, sPAT exhibited smaller variations in NTCP. The result indicates that IMPT is more sensitive to anatomical changes.

**TABLE 5:** Calculated values of TCP and NTCP.

	Primary plans			Non‐adaptive plans		
Structure	sPAT_p_ (*x̄* ± SD)	IMPT_p_(*x̄* ± SD)	*p* value	sPAT_p‐a_(*x̄* ± SD)	IMPT_p‐a_(*x̄* ± SD)	*p* value
CTV (TCP %)	64.99 ± 0.45	65.58 ± 0.58	<0.001^*^	59.16 ± 9.58	57.51 ± 12.12	0.021^*^
Lung (NTCP %)	0.02 ± 0.06	0.04 ± 0.09	<0.001^*^	0.27 ± 0.75	0.31 ± 0.91	0.004^*^
Esophagus (NTCP %)	0.38 ± 0.74	0.40 ± 0.79	0.848	0.46 ± 0.90	0.43 ± 0.79	0.140
Heart (NTCP %)	1.67E‐12 ± 5.29E‐12	5.06E‐12 ± 1.46E‐11	0.230	1.51E‐10 ± 4.47E‐10	1.49E‐10 ± 5.10E‐10	0.296
Spinal Cord (NTCP %)	1.15E‐5 ± 1.60E‐5	9.34E‐5 ± 1.54E‐4	<0.001^*^	4.01E‐5 ± 1.27E‐4	4.90E‐4 ± 1.74E‐3	0.001^*^

*Note*: All TCP and NTCP values are presented as mean ± standard deviation (*x̄* ± SD).

* indicates a statistical significance at *p* < 0.05.

It should be noted that the absolute NTCP values are very low for all OARs in both techniques, and the inter‐technique differences are less than 1%. The NTCP analysis is presented primarily to complement the dosimetric findings, these small differences may have limited clinical relevance.

## 4 DISCUSSION

This study aimed to evaluate the impact of inter‐fractional anatomical changes on the dosimetric and radiobiological performance of sPAT versus IMPT for lung cancer patients. Our results demonstrate that sPAT exhibits a better target coverage and a smaller reduction in TCP against anatomical changes in non‐adaptive planning. This observation aligns with the hypothesis that distributing dose across a larger set of beam directions mitigates sensitivity to density alterations occurring along individual beam trajectories.

PAT integrates proton physical advantages with multi‐angle gantry‐based irradiation.^21^ The greater number of beam angles increases the degrees of freedom in plan optimization, potentially yielding more favorable dose distributions.^22^, ^23^ This can also be seen from the comparison of the primary plans in this study. For tumors exhibiting substantial motion, PAT also demonstrates superior dosimetric advantages.^6^, ^24^, ^25^ These studies reflect the better robustness of PAT to anatomical changes in intro‐fractionation treatment. This study provides supplementary information on the robustness of sPAT and IMPT caused by inter‐fractional anatomical changes.

In this study, a higher spinal cord D_max_ observed in IMPT plans (both primary and non‐adaptive) is attributable to the use of only 3–4 fixed beam angles. For centrally located or mediastinally adjacent tumors, one IMPT beam path may traverse or pass in close proximity to the spinal cord, resulting in concentrated dose deposition. In contrast, sPAT distributes the dose across 13–17 beam directions, substantially diluting the dose contribution from any single direction and thereby reducing the maximum point dose. We acknowledge that with appropriate beam‐angle selection, static‐field IMPT might provide improved spinal cord sparing compared with sPAT.

Chocan et al.^26^ previously reported a lung cancer study, they found that PAT provided slightly better target coverage than IMPT but showed slightly poorer robustness for OARs, particularly the heart and esophagus. This trend also observed in our study. This organ‐specific behavior can be explained by the distinct anatomical positions of these structures relative to the beam geometry. The esophagus is located centrally and posteriorly in the mediastinum, where multiple arc beams converge, leading to a slightly higher dose bath from PAT. Conversely, the heart is positioned anteroinferiorly and is more affected by direct traversal from the limited number of fixed IMPT beams. PAT's angular dispersion reduces the per‐direction contribution to the heart. This distinction explains why the esophagus D_mean_ was slightly higher in PAT while the heart D_mean_ was marginally lower. This suggests that while PAT offers advantages in target coverage, its robustness for OARs located along the beam path may be compromised by density interfaces.

The PAT approach used in this study was a simulated arc plan based on discrete fields rather than true dynamic arc delivery. It should therefore be regarded as an idealized, delivery‐unconstrained, discrete‐beam pseudo‐arc approximation. In clinically deliverable spot‐scanning proton arc therapy (SPArc), gantry rotation is continuous and coupled to spot delivery; the temporal sequence of beam delivery, energy‐layer switching and beam‐on/off timing; per‐angle restrictions on the number of energy layers or spots; and machine‐specific constraints such as the minimum deliverable monitor unit per spot are explicitly modeled in planning and delivery. These delivery constraints reduce the optimization freedom relative to the present sPAT. Consequently, the robustness advantage observed for sPAT may represent an upper bound and may be attenuated under true dynamic arc delivery.

This study has several limitations. sPAT used a simulated arc plan based on discrete fields and was not a true dynamic arc system, so further prospective validation on clinically deliverable arc platforms is warranted. This was a single‐center retrospective analysis with a modest sample size and possible selection bias. In particular, only patients with notable tumor volume changes requiring re‐simulation CT were included, which may overestimate the typical dosimetric impact in the general lung cancer proton therapy population. Tumor shrinkage observed in the photon‐treated cohort may differ from that in proton therapy regimens. In addition, the non‐adaptive plans assumed that the entire 30‐fraction course was delivered on the changed anatomy, which overestimates the actual dosimetric impact because anatomical changes occur gradually. Furthermore, all plans were generated by a single experienced planner. Plan quality may depend on the experience and individual planning choices of the planner, which limits the generalizability of the results. Finally, the study focused primarily on dosimetric comparisons and lacked systematic evaluation of long‐term clinical outcomes. Future multicenter studies incorporating clinically deliverable spot‐scanning proton arc systems are needed to externally validate these findings.

## 5 CONCLUSION

This study evaluated dosimetric and radiobiological outcomes of non‐adaptive sPAT and IMPT plans for 21 lung‐cancer patients under inter‐fractional anatomical changes. sPAT maintained better stability in target coverage and TCP under anatomical changes, reflecting superior robustness against inter‐fractional anatomical changes compared with IMPT.

## AUTHOR CONTRIBUTIONS

Conceptualization: Wenguang Chen and Xianliang Wang. Methodology: Wenguang Chen and Feng Yang. Formal analysis: Wenguang Chen and Feng Yang. Investigation: Wenguang Chen, Fanrui Zeng, Hao Guo, Yuankang Xu, Tianyu Zhang. Resources: Xianliang Wang. Data Curation: Wenguang Chen and Feng Yang. Writing manuscript: Wenguang Chen. Review & editing: Wenguang Chen and Feng Yang. Funding acquisition: Xianliang Wang. All authors have read and agreed to the published version of the manuscript.

## CONFLICT OF INTEREST STATEMENT

The authors declare no conflicts of interest.

## ETHICS STATEMENT

This retrospective study was approved by the Ethics Committee for Medical Research and New Medical Technology of Sichuan Cancer Hospital (Approval No. KY‐2026‐085‐02; Date of approval: June 4, 2026). The requirement for informed consent was waived due to the retrospective nature of the study.

## Data Availability

The data supporting the findings of this study are not publicly available due to ethical restrictions and patient privacy concerns. Data are available from the corresponding author upon reasonable request.
